# Use of Larch, Spruce and Pine Cones as Unconventional Sorbents for Removal of Reactive Black 5 and Basic Red 46 Dyes from Aqueous Solutions

**DOI:** 10.3390/molecules30173614

**Published:** 2025-09-04

**Authors:** Tomasz Jóźwiak, Urszula Filipkowska, Anna Nowicka, Natalia Baranowska

**Affiliations:** Department of Environmental Engineering, University of Warmia and Mazury in Olsztyn, Warszawska St. 117a, 10-957 Olsztyn, Poland; urszula.filipkowska@uwm.edu.pl (U.F.); anna.grala@uwm.edu.pl (A.N.); natalia.baranowska@student.uwm.edu.pl (N.B.)

**Keywords:** cones, larch, spruce, pine, unconventional sorbent, sorption, anionic dyes, Reactive Black 5, cationic dyes, Basic Red 46

## Abstract

This study investigated the sorption properties of the biomass of larch (LaC), pine (PiC) and spruce cones (SpC) in relation to the anionic dye Reactive Black 5 (RB5) and cationic Basic Red 46 (BR46). The scope of the study included the properties of the sorbents (FTIR, SSA, fiber content, elemental analysis C, N, H, pH_PZC_), the effect of pH on the sorption efficiency of the dyes, the sorption kinetics (pseudo-first-order model, second-order model, intraparticle diffusion model) and the maximum sorption capacity of the sorbents (Langmuir 1 and 2 models, Freundlich). The sorption efficiency of RB5 on the sorbents tested was highest at pH 2 and BR46 at pH 6. The pH_PZC_ values determined for LaC, PiC and SpC were 6.86, 7.02 and 7.19, respectively. The sorption equilibrium time depended mainly on the initial dye concentration and ranged from 150 to 180 min for RB5 and from 120 to 210 min for BR46. The sorption capacities (Q_max_) of LaC, PiC and SpC for RB5 were 1.05 mg/g, 1.12 mg/g and 1.61 mg/g, respectively, and for BR46 were 70.53 mg/g, 76.60 mg/g and 96.44 mg/g, respectively. The most efficient sorbent for both dyes was SpC, which was partly related to the high lignin content of the material.

## 1. Introduction

Dyes are chemical compounds that can impart color to other materials. Currently, over 10,000 different types of dyes are known [1,2], and their annual production for the textile, tanning, rubber and paper industries reaches almost 1.6 million tons per year [3,4]. Most of the dyes produced (about 80%) are consumed by the textile industry, which generates almost 70 billion tons of dyed wastewater annually [5,6].

Dyed industrial wastewater is particularly harmful to the environment. The discharge of colored wastewater into natural waters can cause a number of negative changes in local ecosystems. Contamination of water by dyes, even at concentrations as low as 1 mg/L, is clearly visible and disturbs the esthetic appearance of the landscape [7]. Of even greater concern, however, is the limited ability of colored water to transmit sunlight. This limited access to light can lead to reduced photosynthetic efficiency in aquatic autotrophs [1,8]. Some dyes can also react with dissolved oxygen in water [9], which, combined with the inhibition of photosynthesis, can lead to anaerobic conditions in aquatic environments [10]. In addition, a significant proportion of dyes and their partial degradation products can be highly toxic to most aquatic organisms [11,12,13]. Consequently, the contamination of water bodies with colored wastewater can lead to the collapse of the local ecological system [14].

The potential environmental pollution from dyes motivates us to use environmentally friendly yet effective methods to decolorize colored wastewater. Currently, sorption-based technologies [15,16,17] are widely regarded as the most environmentally eco-friendly approaches for removing dyes from wastewater. Sorption is the phenomenon in which molecules of one substance (the sorbate) bind to another (the sorbent). The effectiveness of sorption depends on the type of sorbent used, but also on the type of sorbate, the pH value of the solutions, the temperature and the mixing speed.

Activated carbon [18,19,20] is a commonly used sorbent in wastewater treatment. These materials consist mainly of elemental carbon. They are usually produced from coal [21] or lignocellulosic plant biomass by carbonization and activation processes [22,23,24]. Activated carbon could be also produced from synthetic polymers or waste polymers [25,26]. Activated carbons have a high porosity and a very large specific surface area, often exceeding 1000 m^2^/g [27,28]. Thanks to their highly developed surface area, these materials effectively sorb most industrially used dyes [29,30]. Another advantage of activated carbon is its ability to be regenerated and reused [31,32]. However, their high production costs are a disadvantage that prompts the search for cheaper alternatives [33,34].

Cheap raw materials for the production of unconventional sorbents are usually sought among the waste products of various branches of industry [35,36]. Particularly high hopes are associated with the extraction of sorption materials from waste from the agricultural and food sectors [37,38]. Plant biomass containing lignocellulose has the greatest potential as a raw material for the production of sorbents [39,40]. It is characterized by very high availability in most countries of the world. Its advantage is that it can be used as a sorbent in its original form without the need for complicated pre-treatment. The plant sorbents tested so far include Stems and leaves of crops (e.g., cotton [41], rapeseed [42], wheat [43], pineapple [44] and mango [45]), fruit peels (banana [46], orange, grapefruit, lemon [47]), vegetable peels (potatoes [48], cucumbers [49], garlic [50]), seed coats (e.g., nuts [51], sunflowers [52], cotton [53]). The acidic nature of most biomass-based sorbents makes them particularly useful for the removal of basic (cationic) dyes from aqueous solutions. The efficiency of plant sorbents depends to a large extent on the content of polysaccharides and lignin in their structure [54,55]. Plant materials that could potentially be good sorbents for dyes due to their high content of cellulose, hemicellulose and lignin include conifer cones.

Cones can be obtained in large quantities from coniferous forests. They are also readily available in seed extraction plants, as they are a waste product from the extraction of seeds from coniferous tree tops. In Europe, some of the most numerous trees that produce cones are as follows: Scots pine (*Pinus sylvestris* L.), Norway spruce (*Picea abies* L.) and European larch (*Larix decidua* M.) [56]. The cones of these trees are used in a variety of ways in both industry [57] and agriculture [58]. They are generally used as fuel with a high energy value [59,60], as mulch material, especially for acid-loving plants [61] and to produce acidic compost [62]. As mentioned above, the high total content of polysaccharides and lignins in the dry mass of cones (up to 80–90%) suggests the possibility of using them as an unconventional sorbent for the removal of colorants from aqueous solutions. Theoretically, the type of conifer from which they originate could have a significant impact on the sorption properties of the biomass of cones. However, information on this topic is difficult to find in the scientific literature. Therefore, it seemed advisable to conduct studies comparing the sorption capacity of cones from different popular conifers.

In this article, studies on the sorption properties of the biomass of cones of European larch (LaC), Scots pine (PiC) and Norway spruce (SpC) in relation to colorants popular in the industry are presented: Reactive Black 5 (RB5) and Basic Red 46 (BR46).

## 2. Results and Discussion

### 2.1. Characterization of the Tested Sorbents (FTIR, SSA, Fiber Content, Elemental Analysis C, N, H)

**Figure 1:**
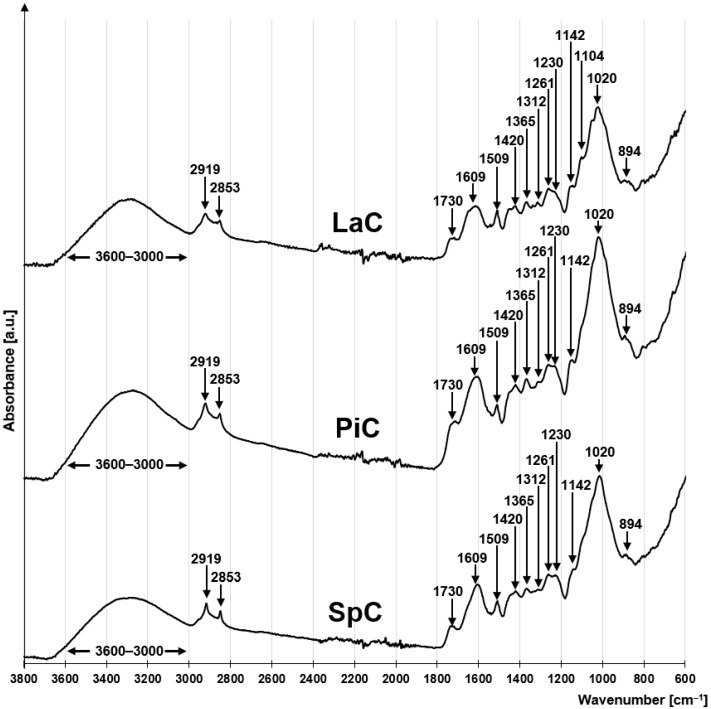
FTIR spectra for LaC, PiC, SpC.

The FTIR spectra of LaC, PiC and SpC are very similar, suggesting that they have a similar chemical composition. All three spectra exhibit a series of peaks characteristic of lignocellulosic plant biomass (Figure 1). The peaks at 1142 cm^−1^, 1104 cm^−1^, 1020 cm^−1^ and 894 cm^−1^ are due to C-O-C glycosidic bonds between the saccharide rings of cellulose and hemicellulose [63,64]. The peaks at 1365 cm^−1^ and 1312 cm^−1^ correspond to the rocking and bending vibrations of the C6 carbon of the pyranose ring of holocellulose [65]. The presence of a peak at 1420 cm^−1^ (shear vibrations -CH_2_) indicates the presence of a crystalline portion of the cellulose [66].

The presence of lignin in the studied materials is associated with a peak at 1230 cm^−1^ indicating vibrations of the syringin ring [67], as well as peaks at 1609 cm^−1^ and 1509 cm^−1^ indicating the presence of vibrations of aromatic lignin structures [67,68]. Another characteristic feature of lignin is the peak at 1730 cm^−1^, which indicates the presence of C=O carbonyl bonds [69,70]. However, it cannot be ruled out that this peak is related to the presence of carboxyl groups, which occur naturally in hemicellulose and in very small amounts in cellulose and lignin. The broad absorption band at 3600–3000 cm^−1^ is attributed to the stretching of the O-H bonds of the hydroxyl functional groups present in cellulose, hemicellulose and lignin [71,72]. The peaks at 2924 cm^−1^ and 2853 cm^−1^ correspond to the asymmetric and symmetric stretching vibrations of -CH_2_ groups, which may belong to aliphatic fragments of holocellulose and lignin structures, but also to terminal protein groups [73,74]. The presence of proteins in the investigated material can be indicated by a small peak at 1261 cm^−1^ corresponding to the stretching of the C-N bond [75].

The specific BET surface areas of the sorbents were very small and amounted to 0.308 m^2^/g, 0.283 m^2^/g and 0.248 m^2^/g for LaC, PiC and SpC, respectively. The average pore size on the surface of LaC, PiC and SpC was 16.7 μm, 20.2 μm and 14.6 μm, respectively, which qualifies the tested materials as macroporous.

The composition of the most important components of the sorbents tested is summarized in Table 1. The cellulose and lignin content increases in the range LaC < PiC < SpC, while in the case of hemicellulose, the situation is reversed and decreases in the series (LaC > PiC > SpC). The high content of hemicellulose, which has acidic functional carboxyl groups in its structure, can increase the acidic character of the sorbent and thus promote the electrostatic binding of basic dyes.

Lignin also has acidic properties and thus also supports the sorption of cationic dyes. However, due to the very small amount of functional carboxyl groups, the acidic property of lignin is much weaker than that of hemicellulose. The acidity of lignin results from the presence of phenolic groups, which can be deprotonated, and aliphatic hydroxyl groups (which exhibit weak acidity) in its structure. 

At a sufficiently low pH value, the phenolic groups and the aliphatic hydroxyl groups of lignin are capable of protonation. Positively charged functional groups are responsible for the electrostatic binding of anionic compounds. It should also be mentioned that lignin, unlike polysaccharides, possesses numerous aromatic structures. This enables it to bind sorbates containing aromatic rings through the formation of strong π−π interactions. Consequently, a higher lignin content in biomass promotes the sorption of both cationic and anionic dyes [76].

However, it should be borne in mind that the sorption of dyes on lignocellulosic bio-mass can occur not only through electrostatic interactions and π-π interactions but also through hydrogen bonds (e.g., with hydroxyl groups of the sorbent) or van der Waals forces [77].

The content of elements (C, N, H) in the cone biomass is very similar (Table 2). The presence of nitrogen is mainly related to the protein content in the biomass. Amino groups derived from amino acids can be important active centers in the sorption of anionic dyes.

The elemental composition of the cone biomass can help to decide on the disposal of the sorbents used. The high carbon content indicates a fairly high calorific value of the materials and suggests the possibility of recovering heat energy, e.g., by incineration in a heating plant. A C/N ratio of approximately 30:1 also indicates the possibility of biomass fermentation and thus energy recovery in the form of biogas [78,79]. Used cone-based sorbents could also be used as a raw material for the production of activated carbon [80].

### 2.2. Effect of pH on the Efficiency of Dye Sorption on LaC, PiC and SpC

**Figure 2:**
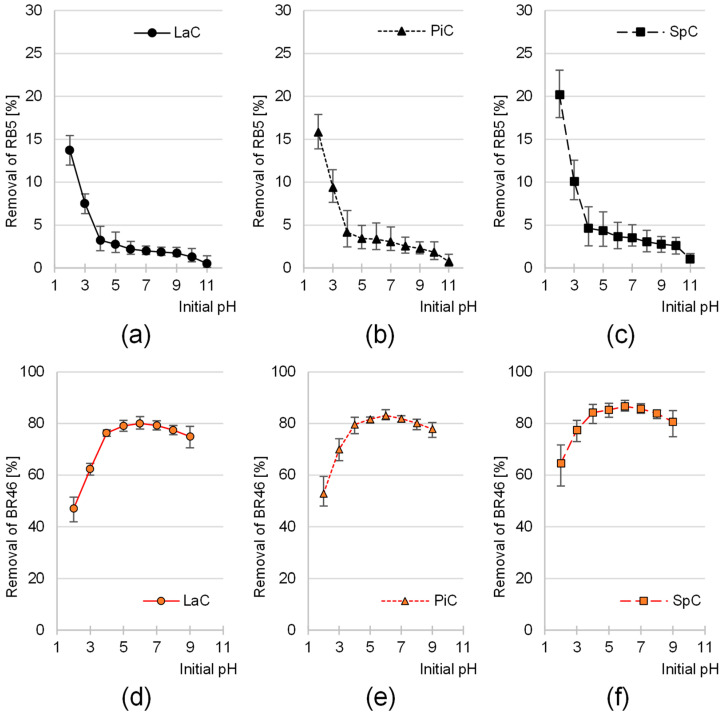
Effect of pH on the sorption efficiency of RB5 onto (**a**) LaC, (**b**) PiC, (**c**) SpC and BR46 onto (**d**) Lac, (**e**) PiC and (**f**) SpC (average + range). Initial conc. of RB5/BR46 = 50 mg/L. Temp. 25 °C.

The sorption efficiency of the reactive anionic dye Reactive Black 5 (RB5) on the biomass of larch (LaC), pine (PiC) and spruce cones (SpC) was highest at pH 2 (Figure 2a–c). The higher the initial sorption pH, the lower the effectiveness of RB5 binding to the cone biomass. The greatest decrease in sorption intensity was observed in the pH range of 2–4, while in the pH range of 4–10, the binding efficiency of the anionic dye RB5 to cones was at a similar, low level. For each sorbent tested, the lowest sorption efficiency was observed at pH 11.

Under low pH conditions with a significant concentration of hydronium ions (H_3_O^+^), the functional groups in the cone biomass were protonated. Presumably, in the polysaccharides (cellulose and hemicellulose), mainly primary aliphatic hydroxyl groups (C6-OH) were protonated, whereas in the lignin structure, phenolic hydroxyl groups could be protonated in addition to aliphatic -OH groups. Due to the presence of proteins in the cone biomass, some amino groups in the amino acids were also protonated.

R-OH + H_3_O^+^ → R-OH_2_^+^ + H_2_O

R-NH_2_ + H_3_O^+^ → R-NH_3_^+^ + H_2_O

The strong positive charge thus generated on the surface of the cone biomass electrostatically attracted the RB5 anions present in the solution, increasing the binding efficiency of this dye.

With increasing pH, i.e., with decreasing concentration of hydronium ions, the efficiency of protonation of the functional groups of the cone biomass decreased. As a result, the total positive charge on the surface of the sorbent decreased, which was reflected in a decreasing ability to sorb the anionic RB5. A characteristic feature of hydroxyl functional groups is that they are only protonated at very low pH values (pH 2–3). When the pH value in the system was increased to pH > 3, practically all hydroxyl groups were present in a non-ionized form. However, a significant proportion of the carboxyl groups were deprotonated at pH > 3. This resulted in numerous negative charges on the surface of the sorbent, which further limited the sorption of the anionic dye. This explains the significant decrease in RB5 binding efficiency on the tested sorbents observed in the pH range of 2–4 (Figure 2a–c).

In the pH range of 4–9, the ionized functional groups on the surface of the cone biomass were probably few amino groups and carboxyl groups.

R-NH_2_ + H_2_O → R-NH_3_^+^ + OH^−^

R-COOH + H_2_O → R-COO^−^ + H_3_O^+^

In a strongly alkaline environment (pH > 10), deprotonation of the aliphatic and phenolic hydroxyl groups of the sorbents can occur in addition to the carboxyl groups.

R-COOH + OH^−^ → R-COO^−^ + H_2_O

R-OH + OH^−^ → R-O^−^ + H_2_O

The strong overall negative charge on the surface of the tested sorbents prevented the diffusion of RB5 onto the surface of the sorbent, further limiting its sorption.

A similar effect of pH on the sorption efficiency of RB5 was also observed in studies on the sorption of this dye on chitin sorbents [81], activated carbon [82], sunflower husks [83] or rapeseed stalks [42].

In contrast to RB5, the sorption efficiency of the cationic dye Basic Red 46 (BR46) on the tested sorbents was lowest at pH 2 and increased with increasing pH (Figure 2d–f). The most significant increase in the binding efficiency of BR46 on the cones was observed with an increase in pH from pH 2 to pH 4. For each of the sorbents tested (LaC, PiC and SpC), the highest sorption efficiency of Basic Red 46 was achieved at an initial pH of 6. A further increase in pH in the system led to a slight decrease in the sorption efficiency of this dye.

Solutions of the dye Basic Red 46 spontaneously decolorize at a pH of >9. This phenomenon is caused by the attack of hydroxyl ions on the electrophilic triazolium group of BR46, which is part of its chromophore system. As a result of this reaction, the system of double bonds in the aromatic ring and the azo group, which is responsible for light absorption, is disrupted. Consequently, the solution becomes bleached. In the series of experiments with BR46, color removal exceeded 90% after a 120-min process at pH 10–11. However, this was not due to sorption, but rather to the degradation of the BR46 chromophore. Therefore, the results of the sorption efficiency of BR46 in the pH range of 10–11 were not considered in Figure 2d–f.

BR46 is alkaline and dissociates in aqueous solution to form colored cations. The mechanism of sorption of BR46 to LaC, PiC and SpC is probably based on ionic interactions between the dye cation and deprotonated functional groups of the sorbent (e.g., carboxyl and hydroxyl groups). Hydrogen bonds, which can form, for example, between the hydrogen atoms of the hydroxyl groups of the sorbent and the nitrogen atoms of the dye, could also be of great importance for the sorption of BR46 on the sorbents tested.

At low pH, the positively charged surface of the cone biomass repelled the BR46 cations electrostatically, resulting in low sorption performance. With increasing pH, the number of protonated functional groups decreased. At the same time, acidic functional groups on the surface of the sorbent (e.g., carboxyl groups) were deprotonated, making it negatively charged. Both processes led to an intensification of the binding of Basic Red 46.

The largest increase in sorption capacity of BR46 on cone biomass observed in the pH range of 2–4 correlated with the largest decrease in sorption capacity of RB5 (Figure 2a–c) and in both cases was caused by the transition of hydroxyl functional groups from the protonated form to the non-ionized form as well as by the transition of carboxyl groups from the non-ionized form to the deprotonated form.

BR46 contains tertiary amino groups which give it a cationic character. At pH above 8, some of the amino groups of BR46 are converted to non-protonated forms and lose their positive charge. The loss of electrostatic charge can lead to a weaker interaction with the sorbent, which explains the decrease in sorption performance of BR46 on pine cones in the pH range of 7–9 (Figure 2d–f).

Similar research results, manifested among other things in a low sorption performance at low pH and at the same time a relatively high dye binding capacity in the pH range of 5–7, were also obtained in studies by other authors. These include experiments on the sorption of Basic Red 46 on pine needles [84], activated carbon [85] and *Paulownia tomentosa* leaves [86].

For each dye, the effect of pH on sorption efficiency on LaC, PiC and SpC was very similar (Figure 2). This suggests that all cone biomass-based sorbents tested have similar chemical properties and a similar dye sorption mechanism.

**Figure 3:**
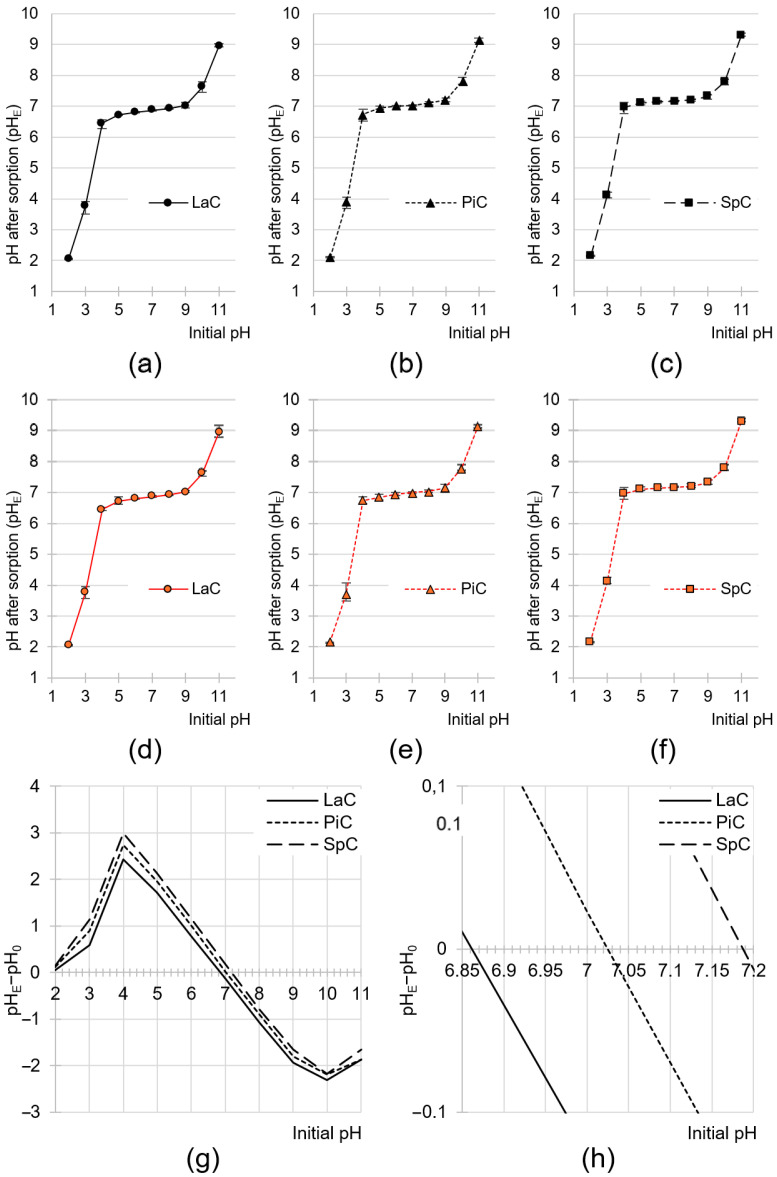
Changes in pH values of the solutions during sorption of RB5 onto (**a**) LaC (**b**) PiC and (**c**) SpC and during sorption of BR46 onto (**d**) LaC (**e**) PiC and (**f**) SpC (average + range). (**g**,**h**) pH_PZC_ of the tested sorbents determined with the “drift” method. Initial conc. of RB5/BR46 = 50 mg/L. Temp. 25 °C.

Cone-based sorbents at a dose of 10 g/L had a marked effect on the pH change in the solution in which sorption was carried out (Figure 3a–f). In the initial pH range of 4–9, the pH of the solution after sorption ranged pH 6.66–7.04 (LaC), pH 6.79–7.12 (PiC) and pH 6.97–7.20 (SpC). The type of dye had no significant effect on the changes in the pH of the solution during sorption.

The pH changes in the solutions are due to the fact that the sorbents have functional groups that are capable of ionization. At low pH, the functional groups capable of protonation absorbed protons from the hydronium ions. As a result, the concentration of H_3_O^+^ ions decreased, leading to an increase in the pH of the solution. In contrast, at a high pH, the groups capable of deprotonation released a proton into the solution, neutralizing the hydroxide anions and lowering the pH of the mixture. The effect of the pH change in the system depends on the ratio of basic groups to acidic groups on the surface of the sorbent. In general, the system always tends to reach a pH value close to the pH_PZC_ value of the sorbent. The pH_PZC_ value (PZC—point of zero charge) is the pH value at which the sorbent reaches zero charge on its surface. In this state, the surface of the sorbent has an equal number of positive and negative charges [87]. The pH_PZC_ value obtained by the drift method was pH_PZC_ = 6.86, pH_PZC_ = 7.02 and pH_PZC_ = 7.19 for LaC, PiC and SpC, respectively (Figure 3g,h). The pH_PZC_ value > 7 obtained for SpC suggests that basic functional groups (e.g., amine) slightly predominate over acidic groups (e.g., carboxyl) on the surface of this sorbent. The opposite is the pH_PZC_ value < 7, which was determined for LaC.

The subsequent stages of the investigation of the sorption properties of LaC, PiC and SpC (described in Section 2.3 and Section 2.4) were carried out at the optimum pH for each dye (pH 2 for RB5 and pH 6 for BR46).

### 2.3. Kinetics of Dye Sorption on LaC, PiC and SpC

**Figure 4:**
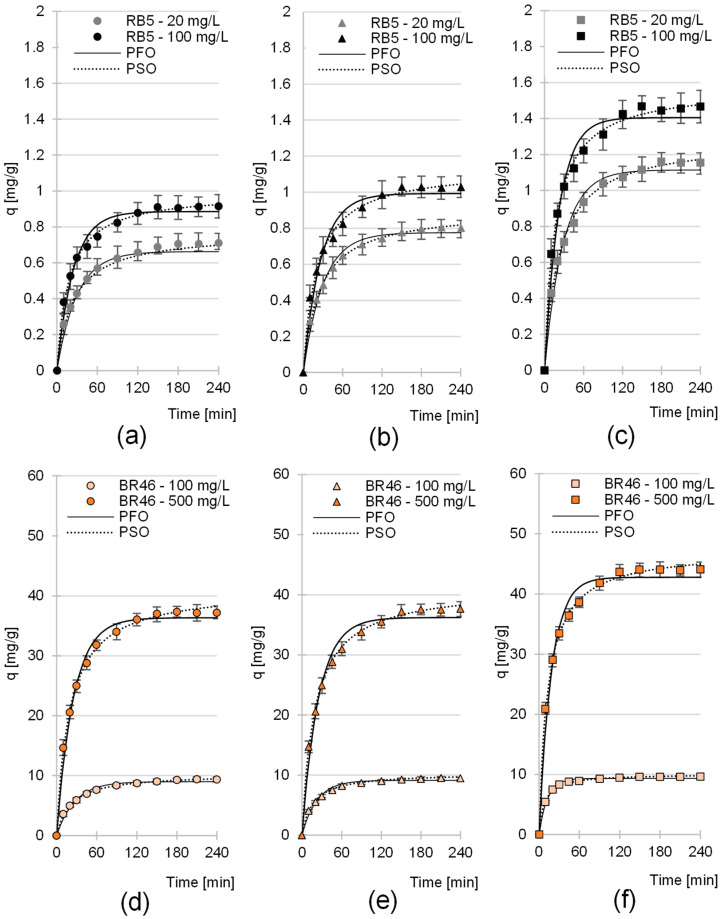
Sorption kinetics of RB5 onto (**a**) LaC, (**b**) PiC and (**c**) SpC and sorption kinetics of BR46 onto (**d**) Lac, (**e**) PiC and (**f**) SpC (average + range). Pseudo-first-order model (PFO) and pseudo-second-order model (PSO). Temp. 25 °C.

The sorption equilibrium time of RB5 on the sorbents tested depended mainly on the initial dye concentration and ranged from 150 min to 180 min (Figure 4a–c, Table 3). The highest dye sorption intensity was recorded at the beginning of the process. After the first 20 min, the amount of dye bound to the RB5 sorbent ranged from 49.8 to 58.3% of q_e_ for LaC, from 50.1 to 54.5% of q_e_ for PiC, and from 52.3 to 59.4% of q_e_ for SpC (q_e_—amount of sorbed dye after reaching sorption equilibrium) (Figure 4a–c).

Similar sorption equilibrium times were also observed in studies on the sorption of Reactive Black 5 onto sorbents such as Canadian goldenrod biomass (150 min) [88], *Eriobotrya japonica* seed hulls (150 min) [89] and rapeseed hulls (180 min) [90].

Apart from the initial dye concentration, the sorption equilibrium time of BR46 also depended on the type of cones. In the LaC and PiC test series, sorption of the tested cationic dye took 180 to 210 min, whereas in the case of SpC, sorption ended within 120–150 min (Table 3). As in the RB5 test series, the highest intensity of binding of BR46 was observed in the first minutes of sorption. After only 20 min of sorption, LaC was able to bind 53.1 to 55.0% q_e_ and PiC 54.8 to 58.1% q_e_. The best results were obtained with SpC, which was able to sorb 66.5–77.9% q_e_ within the first 20 min.

Similar to LaC and PiC, sorption equilibrium times (180–210 min) were also obtained for BR46 in studies on the sorption of this dye on the exoskeletons of flour beetles (180 min) [91], chicken feathers (210 min) [92] and corrugated cardboard (210 min) [76]. Sorption equilibrium times in the range of 120–150 min (as in the case of SpD) were also obtained in studies on the removal of BR46 using sawdust (120 min) [93], coconut shells (120 min) [94], activated charcoal (120 min) [95] and mealworm beetle chitin moltons (150 min) [91].

The shorter sorption equilibrium times obtained in test series with higher initial dye concentrations are probably due to the higher probability of collisions between sorbate molecules and the active sites of the sorbent. Faster saturation of the sorption centers led to faster sorption equilibrium in the system.

The shorter sorption equilibrium times of BR46 on SpC compared to LaC and PiC may be due to the smallest specific surface area. A smaller surface area could have shortened the time to reach sorption equilibrium because it became saturated faster. It is worth noting that despite its smaller specific surface area, SpC has better sorption capacities than LaC and PiC, which will be discussed in the next chapter. The shorter sorption time of the cationic dye on SpC could also be due to the higher lignin content (Table 2, Section 2.1). Lignin has a more complex chemical structure than cellulose or hemicellulose. It contains both aliphatic and aromatic units. In addition to the functional hydroxyl groups, it also contains carbonyl, carboxyl, methoxyl and phenol groups. In lignin, the number of active sites capable of forming hydrogen bonds with nitrogen and hydrogen atoms BR46 is generally higher than in cellulose or hemicellulose [76]. In summary, SpC was characterized by a higher capture rate of dye molecules from solution due to its ability to form a larger number of bonds with cationic dyes, which could ultimately shorten the time required to reach sorption equilibrium. A shorter dye sorption equilibrium time on SpC was not observed for RB5, likely due to a different range of initial RB5 dye concentrations, different initial pH and a distinct sorption mechanism.

The experimental data from studies on the sorption kinetics of RB5 and BR46 on LaC, PiC and SpC were described by pseudo-first-order and pseudo-second-order models (Figure 4, Table 3). In each test series, the pseudo-second-order model showed the best fit to the data obtained. This is a typical result for the sorption of organic dyes on biosorbents.

**Figure 5:**
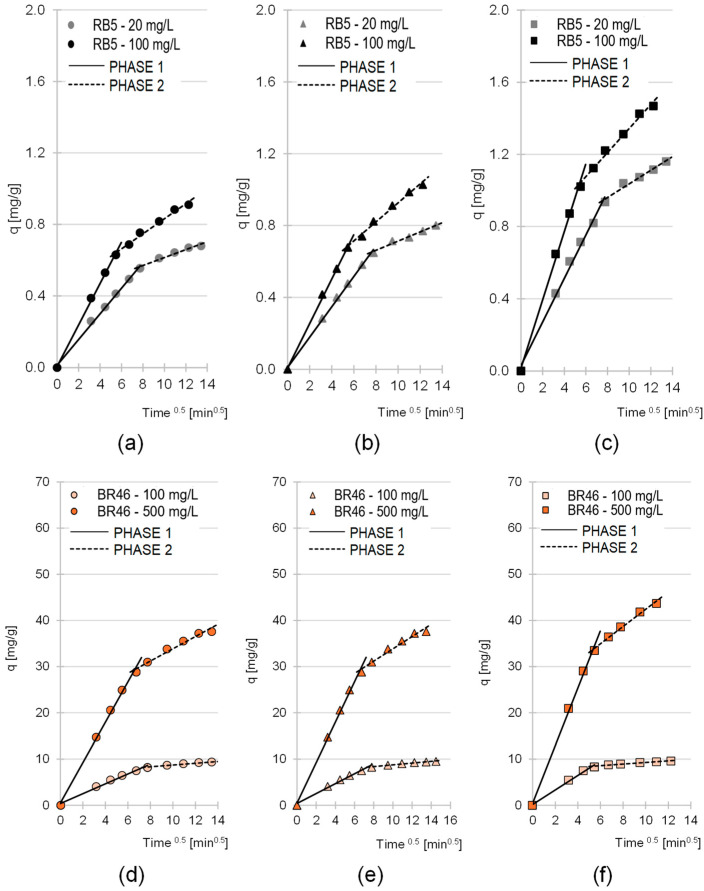
Intraparticle diffusion model for RB5 sorption onto (**a**) LaC, (**b**) PiC and (**c**) SpC and BR46 sorption onto (**d**) Lac, (**e**) PiC and (**f**) SpC (average). Temp. 25 °C.

The experimental data obtained in the studies were also described by an intramolecular diffusion model (Table 4, Figure 5). Analysis of the data presented in the graphs in Figure 5 shows that the sorption of RB5 and BR46 onto LaC, PiC and SpC occurred in two main phases in all test series.

The first phase of sorption was characterized by a high intensity but a short duration. During this phase, the dye particles diffused from the solution onto the surface of the sorbent and then attached themselves to the most accessible active sites. As soon as most of the sorption centers on the surface of the sorbent were saturated, the second phase began. In the second phase, the dye particles penetrated the structure of the material and bound to less accessible active sites in deeper layers of the sorbent. Due to the limited accessibility to the sorption centers and the considerable competition between the sorbate particles, this phase was characterized by a significantly lower intensity than the first phase and generally by a longer duration (k_d1_ and k_d2_ values, phase duration, Table 4). After the last available active sites within the sorbent structure were saturated, the system reached equilibrium.

In all experimental series, the straight line in the first phase of sorption passes through the origin (Figure 5). This means that the resistance to mass transfer of dye molecules from the solution to the outer surface of the sorbent (film diffusion) is practically zero, and the only rate-limiting step for adsorption is intraparticle diffusion.

The q_e,(cal.)_ values determined using the pseudo-second-order model and the k_d1_ and k_d2_ values determined using the intraparticle diffusion model indicate that the sorption efficiency of RB5 on the tested cone biomass-based sorbents is significantly lower than that of BR46 (Table 4). This result is typical for lignocellulosic sorbents and stems from the relatively low number of basic functional groups (such as amino groups) in the tested materials, which are crucial sorption centers for anionic dyes. The kinetic model parameters q_e_, k_d1_ and k_d2_ also indicate a significantly higher efficiency of SpC compared to LaC and PiC. As already mentioned, SpC has a higher lignin content. As explained in point 2.1., higher lignin content favors the sorption of both cationic and anionic dyes. In addition, the higher sorption efficiency of RB5 on SpC compared to LaC and PiC can be explained by a larger amount of typical basic functional groups, which is supported by the highest pH_PZC_ value among the tested sorbents for SpC (pH_PZC_ = 7.19).

### 2.4. Maximum Sorption Capacity of the LaC, PiC and SpC

**Figure 6:**
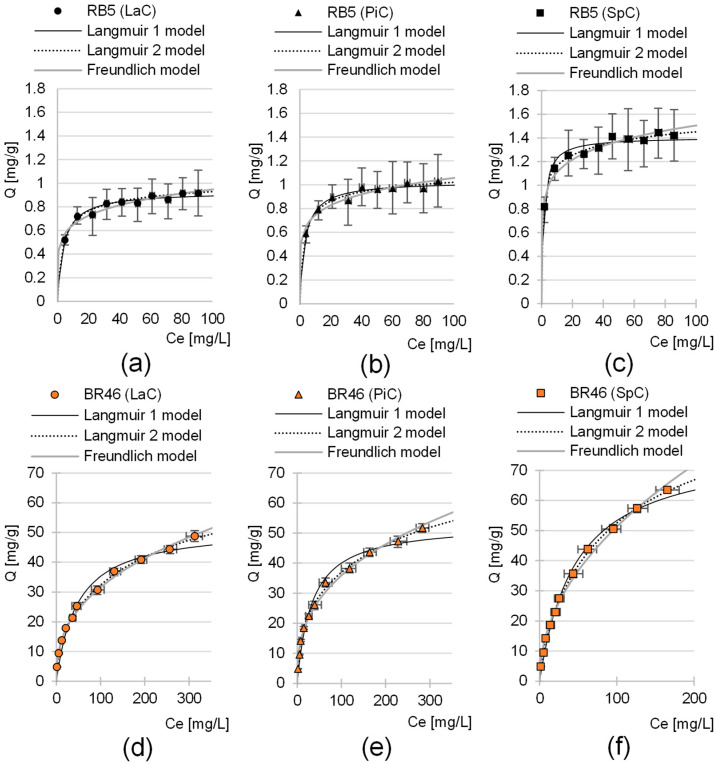
Isotherm of RB5 sorption onto (**a**) LaC, (**b**) PiC and (**c**) SpC and BR46 sorption onto (**d**) Lac, (**e**) PiC and (**f**) SpC (average + range). Langmuir 1, Langmuir 2 and Freundlich models. Temp. 25 °C.

Three adsorption models were used to describe the experimental data from the maximum sorption capacity studies of LaC, PiC and SpC: Langmuir 1 isotherm, Langmuir 2 isotherm and Freundlich isotherm (Table 5). The analysis of the values of the coefficient of determination R^2^ shows that the Langmuir 2 model showed the best fit to the experimental data in each series of investigations (Table 5, Figure 6). This model assumes the existence of at least two types of active centers on the surface of the sorbent, which play an important role in sorption. Presumably, the protonated hydroxyl groups, the protonated amine groups and aromatic rings of lignin that interacted with the aromatic rings of dye (π-π interactions) played the main role in the sorption of RB5 on the biomass of cones. In the case of BR46, in addition to the aromatic rings of lignin, important sorption centers were also deprotonated carboxyl groups and non-ionized hydroxyl groups, which were capable of forming hydrogen bonds with the nitrogen and hydrogen atoms of the dye.

The maximum sorption capacity of LaC, PiC and SpC determined using the Langmuir-2 model was 1.05 mg/g, 1.12 mg/g and 1.61 mg/g for the anionic dye RB5. In the case of the cationic dye BR46, the sorption capacities of LaC, PiC and SpC were significantly higher and amounted to 70.53 mg/g, 76.60 mg/g and 96.44 mg/g, respectively (Table 5). For both RB5 and BR46, the highest sorption capacity was shown for SpC and the lowest for LaC.

As mentioned in Section 2.3, the low sorption performance of RB5 on cone biomass is typical for lignocellulosic sorbents and results from the low amount of typically basic functional groups, which are the most important sorption centers for anionic dyes. The significantly higher molar mass, which is several times higher than that of BR46, could also have had a negative effect on the binding efficiency of RB5 to LaC, PiC and SpC. The larger size of the RB5 molecules could have hindered penetration into the less accessible active sites of the sorbent, ultimately limiting the amount of sorbed dye.

The values of the K_1_ and K_2_ constants determined using the Langmuir-2 model, which are indicators of the affinity of the sorbent for sorbates, are generally much lower in the research series with BR46 than in the series with RB5. This confirms the theory that hydrogen bonding and π-π interactions, which are much weaker than ionic interactions between ionized functional groups of the sorbent and sorbate, play an important role in the sorption of BR46 onto cone biomass. This suggests that cone-based sorbents will show the greatest benefit at high BR46 concentrations in wastewater.

The sorption efficiency series (determined based on Q_max_) as well as the affinity degree series of the tested sorbents for dyes (based on constants K_1_, K_2_, Table 5) are as follows: LaC < PiC < SpC. The result obtained is quite interesting, especially considering that the specific surface area series of the sorbents tested is reversed (SpC < PiC < LaC). This could indicate that, in the case of cone-based materials, the specific surface area is not the best indicator of the efficiency of the biosorbents. The composition of the sorbent material seems to be more important, in particular the type and density of functional groups acting as active sites. The higher sorption capacity of SpC compared to LaC and PiC is probably due to the higher lignin content in the material, which favors the sorption of both cationic and anionic dyes, as mentioned in the previous chapters. In addition, the binding efficiency of RB5 on SpC is the highest, which is also due to the highest content of basic functional groups, as confirmed by the highest pH_PZC_ value among the sorbents tested (Section 2.2, Figure 3g,h).

Table 6 shows the sorption capacities of various unconventional sorbents and activated carbons in relation to RB5 and BR46.

The sorption capacity of SpC, PiC and LaC in relation to RB5 is relatively low and similar to that of biosorbents such as coconut shells, pumpkin seed shells or macadamia nut shells. Much better biosorbents for RB5 were materials such as newsprint, rapeseed hulls or wheat straw. The better sorption capacity of the mentioned biosorbents towards RB5 compared to cone-based materials could result from the higher concentration of basic functional groups on the surface of these biosorbents and their much larger specific surface area. However, the lignocellulosic sorbents did not achieve the efficiency of activated carbon-based materials (Table 6).

The sorption performance of BR46 on SpC, PiC and LaC is higher than most of the biosorbents tested so far (sawdust, seed husks, fruit peels). The sorption capacities of the sorbents tested with respect to BR46 were even higher than those of some activated carbon-based materials (Table 6). This suggests the possibility of using the biomass of cones for the treatment of wastewater containing cationic dyes.

When researching unconventional sorbents, it is advisable to consider the possibility of multiple uses of sorbent materials. According to the authors, the regeneration of sorbents based on cones is not economically justified due to the low price and wide availability of the raw material. When reusing biosorbents, the dyes would have to be desorbed, which would require expensive chemical reagents. A bigger problem, however, is the fact that the regeneration of this type of sorbent would produce colored wastewater that would have to be purified or disposed of. As mentioned in Section 4.1, a better solution seems to be to dry the spent sorbents and then co-incinerate them, e.g., in a heating plant, which would result in energy recovery from the materials. An alternative solution is to ferment the used biosorbents and produce biogas. It has been proven that dyes such as BR46, even in large quantities, do not significantly limit methanogenesis [118]. Used cone-based biosorbents could also be used to produce good quality activated carbon, which could also be used for wastewater treatment.

## 3. Materials

### 3.1. Cones of Conifers

In this work, cones of Scots pine (*Pinus sylvestris* L.), Norway spruce (*Picea abies* L.) and European larch (*Larix decidua* M.) were used for the production of sorbents. The cones were supplied by a seed extraction plant in Jedwabno (Warmian-Masurian Voivodeship, Poland). The cones were subjected to a thermal extraction process, which took place for 12 h at a temperature of 60 °C.

### 3.2. Dyes

**Figure 7:**
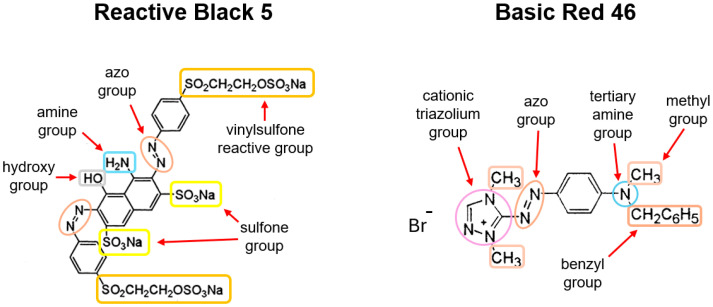
Chemical structure of Reactive Black 5 and Basic Red 46 dyes.

The anionic reactive dye Reactive Black 5 (RB5) and the cationic dye Basic Red 46 (Figure 7) were used for the study. The dyes come from the dye factory “Boruta ZACHEM Kolor” in Zgierz (Poland). Table 7 contains the manufacturer’s information on the dyes.

### 3.3. Chemical Reagents

The following chemical reagents were used in the study:

- Hydrochloric acid (HCl)—37%—(solution pH correction),
- Sodium hydroxide (NaOH) > 99.9%—micropellets—(solution pH correction),
- Acetone (C_3_H_6_O, >99.5%)—cleaning the diamond crystal in the ATR attachment of the spectrometer,
- Buffer solutions (pH 4 ± 0.05/pH 7 ± 0.05/pH 10 ± 0.05)—calibration of the pH meter.

All chemical reagents used were purchased from POCH S.A., Gliwice, Poland, and were of p.a. (analytical purity) grade or higher.

### 3.4. Laboratory Equipment

The following laboratory equipment was used during the study:

- HI 110 pH meter (HANNA Instruments, Olsztyn, Poland)—for measuring and correcting the pH value of solutions;
- Laboratory shaker SK-71 (JEIO TECH, Daejeon, Republic of Korea)—for dye sorption studies;
- Multi-Channel Stirrer MS-53M (JEIO TECH, Daejeon, Republic of Korea)—for dye sorption studies;
- UV-3100 PC spectrophotometer (VWR Spectrophotometer, VWR International LLC., Mississauga, ON, Canada)—for analyzing dye concentration in solutions;
- Knife grinder LMN-100—(Testchem, Radlin, Poland)—for crushing cones.
- FT/IR-4700LE FT-IR spectrometer with single reflection ATR attachment (JASCO International, Tokyo, Japan)—for the generation of FTIR spectra of sorbents;
- FLASH 2000 Analyzer (Thermo Scientific, Waltham, MA, USA)—for elemental analysis and measurement of carbon, hydrogen and nitrogen contents.
- ASAP 2020 (Micromeritics, Norcross, GA, USA)—for measuring the porosity and surface area of sorbents.
- Ankom 220 Fiber Analyzer—(ANKOM Technology, Macedon, NY, USA)—for measuring the content of fiber in cones.
- Batch grinder A 10 basic—(IKA-Werke GmbH & Co KG, Staufen, Germany)—for grinding cones (for fiber content analysis).

## 4. Methodology

### 4.1. Preparation of Cone-Based Sorbents

The cones were crushed in a knife mill with a 3 mm sieve. The resulting crushed cone biomass with a fraction ≤ 3 mm was then sieved through a sieve with a mesh diameter of 2 mm. The fraction that remained on the sieve (2–3 mm) was washed with a large amount of deionized water and then dried in a laboratory dryer (105 °C). The dried biomass of the cones constituted a sorbent ready for testing. The sorbents produced in this way, based on larch cones (LaC), pine cones (PiC) and spruce cones (SpC), were stored in sealed polyethylene containers.

### 4.2. Studies on the Characteristics of Sorbents

#### 4.2.1. Performing FTIR Spectra of the Sorbents

FTIR spectra of the cone biomass were performed using an FT/IR-4700LE FT-IR spectrometer with a single reflection ATR diamond crystal. The scanning range of the samples was infrared with a wavelength range of 4000–400 cm^−1^ and the resolution of the spectra was 1 cm^−1^. The final appearance of the spectrum of each sample was created from the average values of 64 measurements. The baseline was corrected before each measurement.

#### 4.2.2. Measurement of the Specific Surface Area of the Sorbents

The BET surface area of the sorbents tested was measured with an ASAP 2020 instrument (Micromeritics, Norcross, GA, USA) using the low temperature nitrogen adsorption/desorption method. Prior to the measurements, the sorbents were degassed at 100 °C for 4 h.

#### 4.2.3. Analysis of the Fiber Content

The cellulose, hemicellulose and lignin content was determined according to the method proposed by Van Soest et al. [119]. A semi-automatic Ankom 220 Fiber Analyzer (ANKOM Technology, Macedon, NY, USA) was used for chemical fractionation with detergents. The method analyzed the compactness of the neutral-detergent fiber (NDF), acid-detergent fiber (ADF) and acid-detergent lignin (ADL) fractions. Preparation of the samples for analysis consisted of grinding in an A 10 base mill using a sieve with an opening diameter of 1.0 mm. The measurements were performed in 3 replicates and the results were averaged.

#### 4.2.4. Elemental Analysis (C, N, H Content)

A Flash 2000 Elemental Analyzer (Thermo Scientific, Waltham, MA, USA) operating in CNH mode was used to quantify the elemental contents. The instrument used the method of dynamic full combustion of the samples in a reduction-oxidation furnace with electronically controlled temperature (left furnace 950 °C, right furnace 840 °C). The weight of the prepared samples was between 100 and 150 mg. The analyses were performed in 3 replicates and the results were averaged.

### 4.3. Studies on the Influence of pH on the Efficiency of Dye Sorption

Dye solutions (RB5 or BR46) with a concentration of 50 mg/L and a pH value between 2 and 11 were prepared in volumetric flasks (capacity 1000 mL). The tested cone-based sorbent was weighed into a series of beakers (1000 mL capacity) in quantities of 10.0 g dry weight. Magnetic stirrers (8 × 40 mm) and the previously prepared dye solutions were then added to the beakers. The beakers were placed on a multistage magnetic stirrer set to 200 rpm. After 120 min of sorption, samples (10 mL) were removed from the beakers using an automatic pipette and transferred to previously prepared test tubes to later analyze the dye concentration remaining in the solution. The pH value of the solutions after sorption was also measured.

### 4.4. Studies on the Sorption Kinetics of the Dye

In 1000 mL volumetric flasks, dye solutions were prepared at concentrations of 20 and 100 mg/L (for RB5) or 100 and 500 mg/L (for BR46), each at the most favorable sorption pH. (This pH was determined based on previous studies, the methodology for which is presented in Section 4.3.) Into 1000 mL beakers, 10.0 g dry weight of the tested sorbent was weighed, and magnetic stir bars (8 × 40 mm) were added. Subsequently, the prepared dye solutions were added to the beakers, and the beakers were then placed on a multi-position magnetic stirrer (200 rpm). At specified time intervals of 0, 10, 20, 30, 45, 60, 90, 120, 150, 180, 210, 240, 270 and 300 min, 5 mL solution samples were collected from each beaker using an automatic pipette into pre-prepared test tubes for later analysis of dye concentration.

### 4.5. Studies on the Maximum Sorption Capacity of the Tested Sorbents

Dye solutions with concentrations of 10; 20; 30; 40; 50; 60; 70; 80; 90; 100 mg/L (for RB5) or 50; 100; 150; 200; 250; 300; 400; 500; 600; 800 mg/L (for BR46) were prepared in volumetric flasks (capacity 1000 mL). The solutions had a pH that was most favorable for dye sorption (determined based on tests whose methodology is presented in Section 4.3). Then 10.0 g of the dry sorbent was weighed into beakers (1000 mL capacity). In the next step, magnetic stirrers (8 × 40 mm) and the previously prepared dye solutions were added to the beakers. The beakers were then placed on a multistage magnetic stirrer for the duration of the sorption equilibrium (the time was determined based on tests, the methodology of which is presented in Section 4.4). After this time, samples of the solutions (10 mL) were taken with a pipette into prepared empty test tubes to later determine the dye concentration remaining in the solution.

### 4.6. Comments on Section 4.3, Section 4.4 and Section 4.5

- The cone-based sorbents (LaC, PiC, SpC) were weighed into beakers with an accuracy of 0.01 g using a precision laboratory balance.
- The different initial concentrations of the dyes RB5 and BR46 used in the test series in Section 4.4 and Section 4.5 are due to the large differences in the sorption performance of anionic and cationic dyes on the sorbents tested. However, at least one concentration common to both dyes was used in each phase of the study so that the sorption efficiency of RB5 and BR46 could be compared.
- The stirring speed set on the magnetic stirrer ensured an even distribution of the sorbent throughout the volume of the solution.
- The concentration of the dye remaining in the solution was determined spectrophotometrically using a UV-VIS spectrophotometer with a quartz cuvette with an optical path length of 10 mm. The calibration curves used by the spectrophotometer to determine the dye concentrations were generated at λmax for the concentration range 0.0–50.0 mg/L. If necessary, the dye solutions were diluted with deionized water.
- The sorption capacities of the individual sorbent (LaC, PiC, SpC) were tested for both the dyes RB5 and BR46. All test series were carried out in triplicate. The results of the analyses were averaged.
- A constant temperature of 25 °C was maintained in the laboratory during the analyses.

### 4.7. Computation Methods

The content of structural polymers in the cell wall was calculated using Equations (1)–(3):

Cellulose [%] = ADF-ADL(1)

Hemicellulose [%] = NDF-ADF(2)

Lignin [%] = ADL(3)

ADF—acid detergent fiber; ADL—acid detergent lignin; NDF—neutral detergent fiber.

The amount of dye bound to the sorbents (LaC, PiC, SpC) was calculated using Equation (4):

(4) $$Q_{S}=(C_{0}-C_{S})\times \frac{V}{m}$$

Q_S_—mass of sorbed dye [mg/g];
C_0_—initial dye concentration [mg/L];
C_S_—dye concentration after sorption [mg/L];
V—volume of dye solution [L];
m—mass of used sorbent [g].

The kinetics of sorption of RB5 and BR46 on LaC, PiC and SpC were described by pseudo-first-order (5), pseudo-second-order (6) and intramolecular diffusion (7) models

(5) $$q=q_{e}\times (1-e^{\left(-k_{1}\times t\right)})$$

(6) $$q=\frac{\left(k_{2}\times {q_{e}}^{2}\times t\right)}{\left(1+k_{2}\times q_{e}\times t\right)}$$

(7) $$q=k_{id}\times t^{0.5}$$

q—instantaneous value of sorbed dye [mg/g];
q_e_—the amount of dye sorbed at the equilibrium state [mg/g];
t—time of sorption [min];
k_1_—pseudo-first-order adsorption rate constant [1/min];
k_2_—pseudo-second-order adsorption rate constant [g/(mg × min)];
k_id_—intraparticular diffusion model adsorption rate constant [mg/(g × min0.5)].

Experimental data from studies on the maximum sorption capacity LaC, PiC and SpC for RB5 and BR46 were described by three common sorption models: Langmuir isotherm 1 (8), Langmuir isotherm 2 (9) and Freundlich isotherm (10).

(8) $$Q=\frac{\left(Q_{max}\times K_{C}\times C\right)}{\left(1+K_{C}\times C\right)}$$

(9) $$Q=\frac{\left(b_{1}\times K_{1}\times C\right)}{\left(1+K_{1}\times C\right)}+\frac{\left(b_{2}\times K_{2}\times C\right)}{\left(1+K_{2}\times C\right)}$$

(10) $$Q=K\times C^{\frac{1}{n}}$$

Q—mass of sorbed dye [mg/g];
Q_max_—maximum sorption capacity in Langmuir equation [mg/g];
b_1_—maximum sorption capacity of sorbent (type I active sites) [mg/g];
b_2_—maximum sorption capacity of sorbent (type II active sites) [mg/g];
K_C_—constant in Langmuir equation [L/mg];
K_1_,K_2_—constants in Langmuir 2 equation [L/mg];
K—the equilibrium sorption constant in Freundlich model;
n—Freundlich equilibrium constant;
C—concentration of the dye remaining in the solution [mg/L].

## 5. Conclusions

The biomass of larch, pine and spruce cones can be an efficient sorbent for cationic dyes such as BR46. The sorption capacities of LaC, PiC and SpC are similar to those of some types of activated carbons, suggesting that they can be a substitute for conventional sorbents in systems for decolorization of wastewater contaminated with cationic dyes. The low sorption efficiency of RB5 on the tested materials indicates their low suitability for removing anionic dyes.

Among the sorbents tested, SpC shows the highest suitability for the removal of BR46 and RB5. This is probably due to the high content of lignin, whose presence in the sorbent is advantageous for both cationic and anionic dyes. The sorption of RB5 on SpC is also favored by the relatively high content of basic functional groups, as evidenced by the highest pH_PZC_ value among the tested sorbents.

The sorption efficiency of RB5 and BR46 on LaC, PiC and SpC is highly dependent on the pH of the solution. The binding efficiency of RB5 on the tested sorbents is highest at pH 2, while for BR46, it is at pH 6.

The equilibrium sorption time of dyes on the biomass of cones is 150 to 180 min for RB5 and 120 to 210 min for BR46 and depends mainly on the initial concentration of the dye in the solution. In addition, shorter sorption times of BR46 on SpC were found compared to LaC, PiC and SpC, which was explained by the smallest specific surface area and the highest lignin content.

The sorption of RB5 and BR46 on the tested sorbents occurs in two main phases. The first phase is characterized by a high intensity but a relatively short duration, while the second phase lasts longer but is characterized by a lower efficiency.

At least two types of active sites play an important role in the sorption of RB5 and BR46 onto LaC, PiC and SpC. This is evident from the better fit of the Langmuir 2 model to the experimental data obtained compared to the Langmuir 1 model. Presumably, protonated hydroxyl groups, protonated amino groups and aromatic rings of lignin (π-π interactions) play an important role in the sorption of RB5 to the biomass of cones. In the case of BR46, in addition to the aromatic structures of lignin, deprotonated carboxyl groups and non-ionized hydroxyl groups were also important.

## Figures and Tables

**Table 1:** The composition of the most important components of the sorbents.

Component	LaC	PiC	SpC
Cellulose [%]	34.62 ± 1.27	38.61 ± 1.10	44.17 ± 1.24
Hemicellulose [%]	40.08 ± 1.24	39.12 ± 1.03	29.80 ± 1.06
Lignin [%]	17.61 ± 0.98	17.73 ± 1.15	19.90 ± 1.07
Other (proteins, minerals) [%]	7.69 ± 1.14	4.54 ± 0.86	6.13 ± 0.98

**Table 2:** Carbon, nitrogen and hydrogen contents in the cones.

Element	LaC	PiC	SpC
C [%]	45.58 ± 0.49	47.65 ± 0.40	44.11 ± 0.10
N [%]	1.37 ± 0.03	1.42 ± 0.04	1.41 ± 0.02
H [%]	6.28 ± 0.07	6.17 ± 0.07	6.55 ± 0.21

**Table 3:** Kinetic parameters of RB5 and BR46 sorption onto LaC, PiC and SpC, determined from the pseudo-first-order and pseudo-second-order models (based on the average of three measurements) + sorption equilibrium time.

Sorbent	Dye	Dye Conc.	Pseudo-First-Order Model			Pseudo-Second-Order Model			Exp. Data	Equil. Time
			k_1_	q_e,(cal.)_	R^2^	k_2_	q_e,(cal.)_	R^2^	q_e, exp_	
		[mg/L]	[1/min]	[mg/g]	-	[g/mg×min]	[mg/g]	-	[mg/g]	[min]
LaC	RB5	20	0.0338	0.66	0.9844	0.0551	0.77	0.9966	0.68	180
		100	0.0410	0.89	0.9774	0.0578	0.99	0.9968	0.91	150
	BR46	100	0.0372	8.99	0.9841	0.0046	10.30	0.9988	9.39	210
		500	0.0398	36.35	0.9888	0.0013	41.34	0.9987	37.36	180
PiC	RB5	20	0.0334	0.78	0.9859	0.0452	0.90	0.9980	0.80	180
		100	0.0376	0.99	0.9731	0.0433	1.13	0.9948	1.03	150
	BR46	100	0.0448	9.13	0.9853	0.0060	10.35	0.9978	9.50	210
		500	0.0397	36.23	0.9857	0.0013	41.35	0.9994	37.58	180
SpC	RB5	20	0.0350	1.11	0.9820	0.0344	1.28	0.9973	1.15	180
		100	0.0450	1.41	0.9742	0.0394	1.58	0.9962	1.46	150
	BR46	100	0.0806	9.36	0.9935	0.0132	10.08	0.9971	9.62	150
		500	0.0544	42.80	0.9839	0.0017	47.33	0.9989	43.68	120

**Table 4:** Rate constants of RB5 and BR46 diffusion determined from a simplified intraparticle diffusion model.

Sorbent	Dye	Dye Conc.	Phase I			Phase II		
			k_d1_	Time	R^2^	k_d2_	Time	R^2^
		[mg/L]	[mg/(g × min^0.5^)]	[min]	-	[mg/(g × min^0.5^)]	[min]	-
LaC	RB5	20	0.0716	60	0.9960	0.0221	120	0.9649
		100	0.1156	30	0.9982	0.0422	120	0.9851
	BR46	100	0.9962	60	0.9913	0.2603	150	0.9602
		500	4.3686	45	0.9961	1.2500	135	0.9344
PiC	RB5	20	0.0845	60	0.9978	0.0255	120	0.9820
		100	0.1240	30	0.9820	0.0530	120	0.9891
	BR46	100	1.0687	60	0.9858	0.1893	150	0.9704
		500	4.3684	45	0.9961	1.3287	135	0.9710
SpC	RB5	20	0.1205	60	0.9921	0.0376	120	0.9732
		100	0.1886	30	0.9960	0.0665	120	0.9854
	BR46	100	1.5650	30	0.9907	0.1773	120	0.9620
		500	6.2214	30	0.9961	1.8665	90	0.9873

**Table 5:** Constants determined from Langmuir 1, Langmuir 2 and Freundlich models.

Sorbent	Dye	Langmuir 1 Model			Langmuir 2 Model						Freundlich Model		
		Q_max_	K_c_	R^2^	Q_max_	b_1_	K_1_	b_2_	K_2_	R^2^	k	n	R^2^
		[mg/g]	[L/mg]	-	[mg/g]	[mg/g]	[L/mg]	[mg/g]	[L/mg]	-	-	-	-
LaC	RB5	0.93	0.2462	0.9476	1.05	0.23	0.0141	0.82	0.3379	0.9676	0.4419	0.17	0.9279
	BR46	51.99	0.0212	0.9701	70.53	54.31	0.0046	16.22	0.1760	0.9975	5.3232	0.39	0.9941
PiC	RB5	1.03	0.3008	0.9469	1.12	0.23	0.0175	0.89	0.4438	0.9612	0.5248	0.15	0.9112
	BR46	53.25	0.0302	0.9706	76.60	53.67	0.0041	22.93	0.1400	0.9976	6.6653	0.37	0.9890
SpC	RB5	1.41	0.6734	0.9300	1.61	0.42	0.0181	1.19	1.1512	0.9825	0.8181	0.13	0.9219
	BR46	78.24	0.0214	0.9908	96.44	82.64	0.0091	13.80	0.1888	0.9991	5.4792	0.49	0.9931

**Table 6:** Comparison of the sorption capacity of sorbents in relation to RB5 and BR46.

Dye	Sorbent	Q_max_ [mg/g]	pH of Sorption	Time of Sorption [min]	Source
RB5	Coconut shells	0.8	2	60	[94]
	Pumpkin seed husks	1.0	3	60	[96]
	Larch cones	1.0	2	150	This work
	Sunflower biomass	1.1	2	210	[97]
	Pine cones	1.1	2	150	This work
	*Macadamia* seed husks	1.2	3	510	[98]
	Spruce cones	1.6	2	150	This work
	Canadian goldenrod biomass	2.3	3	150	[88]
	Cotton fibers	2.7	3	240	[99]
	Sunflower seed husks	2.9	3	210	[83]
	Buckwheat husks	4.4	3	300	[100]
	Compost	4.8	3	180	[101]
	Newspaper	7.1	3	120	[102]
	Cotton seed husks	12.9	2	30	[103]
	*Eriobotrya japonica* seed husks	13.8	3	150	[89]
	Beech sawdust	13.9	3	1440	[104]
	Rapeseed husks	15.2	3	180	[90]
	Wheat straw	15.7	3	195	[43]
	Activated carbon from wood (walnut)	19.3	5	400	[105]
	Activated carbon from palm shells	25.1	2	300	[106]
	Activated carbon from bamboo	39.0	2	60	[107]
	Activated carbon (powder)	58.8	-	-	[108]
BR46	Chicken feathers	4.1	5	210	[92]
	Wood sawdust	19.2	-	120	[93]
	Office paper (used)	19.6	6	90	[76]
	Natural sugarcane stalks powder	21.0	7.2	60	[109]
	Bone meal	24.6	6	90	[110]
	Activated carbon	26.4	5	240	[111]
	Nut sawdust	30.1	7	-	[112]
	Exoskeletons of mealworm	31.5	6	180	[91]
	Biochar from *Chrysanthemum morifolium* straw	32.3	10	60	[113]
	*Paulownia tomentosa* tree leaves	43.1	8	72	[86]
	ROW 08 activated carbon	45.0	8	60	[85]
	Lemon peels	54.0	6	240	[47]
	Spent green tea leaves	58.0	6	240	[114]
	Rapeseed husks	59.1	6	180	[115]
	*Cerbera odollam* biomass activated carbon	65.7	7	90	[116]
	Coconut shells	68.5	6	120	[94]
	Larch cones	70.5	6	180	This work
	Pine leaves	71.9	6	75	[84]
	Pine cones	76.6	6	180	This work
	Spruce cones	96.4	6	120	This work
	Activated carbon “Chemviron”	106.0	7.4	120	[95]
	Palm bio-waste-derived activated carbon	263.2	5.5	50	[117]

**Table 7:** Characteristics of dyes used in this study.

Dye Name	Reactive Black 5 (RB5)	Basic Red 46 (BR46)
Chemical formula	C_26_H_21_N_5_Na_4_O_19_S_6_	C_18_H_21_BrN_6_
Molecular weight	991 g/mol	321.4 g/mol
Dye type	anionic (reactive)	cationic (basic)
Dye class	double azo dye	single azo dye
λ_max_	600 nm	530 nm
Uses	dyeing of cotton, viscose, wool	dyeing of leather, paper, wool and acrylic fibers
Other trade names	Begazol Black B; Remazol Black B	Anilan Red GRL, Cationic Red X-GRL
Purity of the commercial product	dye content 70%	dye content 80%

## Data Availability

The data presented in this study are available on request from the corresponding author.
